# First positronium lifetime imaging with scandium-44 on a long axial field-of-view PET/CT

**DOI:** 10.3389/fnume.2025.1648621

**Published:** 2025-11-20

**Authors:** Lorenzo Mercolli, William M. Steinberger, Pascal V. Grundler, Anzhelika Moiseeva, Saverio Braccini, Maurizio Conti, Paweł Moskal, Narendra Rathod, Axel Rominger, Hasan Sari, Roger Schibli, Robert Seifert, Kuangyu Shi, Ewa Ł. Stepień, Nicholas P. van der Meulen

**Affiliations:** 1 Department of Nuclear Medicine, Inselspital, Bern University Hospital, University of Bern, Bern, Switzerland; 2 ARTORG Center for Biomedical Engineering Research, University of Bern, Bern, Switzerland; 3 Albert Einstein Center for Fundamental Physics (AEC), Laboratory for High Energy Physics (LHEP), University of Bern, Bern, Switzerland; 4 Siemens Medical Solutions USA, Inc., Knoxville, TN, United States; 5 Center for Radiopharmaceutical Sciences, PSI Center for Life Sciences, Villigen-PSI, Switzerland; 6 Faculty of Physics, Astronomy and Applied Computer Science, Jagiellonian University, Krakow, Poland; 7 Centre for Theranostics, Jagiellonian University, Krakow, Poland; 8 Siemens Healthineers International AG, Zürich, Switzerland; 9 Department of Chemistry and Applied Biosciences, ETH Zurich, Zurich, Switzerland; 10 Laboratory of Radiochemistry, PSI Center for Nuclear Engineering and Sciences, Villigen-PSI, Switzerland

**Keywords:** scandium-44, long axial field-of-view PET/CT, positronium, positronium lifetime imaging, NEMA phantom

## Abstract

**Purpose:**

The physical properties of ^44^Sc, combined with its imminent clinical application, position it as a prime candidate for *in vivo* positronium lifetime imaging. In this study, we investigate the count statistics for ortho-positronium (oPs) measurements with ^44^Sc on a commercial long-axial field-of-view (LAFOV) PET/CT.

**Method:**

A NEMA image quality phantom was filled with 41.7 MBq of ^44^Sc dissolved in water and scanned on a LAFOV PET/CT. Three-photon events were identified using a prototype feature of the scanner and dedicated software. The lifetime of oPs was determined in the phantom spheres and in 4×4×4 mm^3^ voxels.

**Results:**

All measured oPs lifetimes are compatible, within the uncertainties, with the literature values for water. The oPs lifetime is 2.65±0.50, 1.39±0.20 and 1.76±0.18 ns in the three smallest spheres of the phantom and 1.79±0.57 ns for a single voxel in the central region of the largest sphere. The relative standard deviation in the background regions of the time difference distributions, i.e., for time differences smaller than −2.7 ns, is above 20%—even for voxels inside the phantom spheres.

**Conclusions:**

Despite the favorable physical properties of ^44^Sc, the count statistics of three-photon events remains a challenge. The high prompt-photon energy causes a significant amount of random three-photon coincidences with the given methodology and, therefore, increases the statistical uncertainties on the measured oPs lifetime.

## 1 Introduction

Investigating the lifetime of ortho-positronium (oPs), the spin-1 state of an electron-positron bound system, has offered valuable insights into the structural properties of matter for decades (1–8). More recently, the medical community has shown interest in measuring oPs lifetimes in human tissue (9–12). So-called *oPs lifetime imaging*, i.e., constructing a three-dimensional image of the human body with the oPs lifetime as voxel value (13), has the potential to provide diagnostic information about the tissue microenvironment, in particular oxygenation levels, that is currently unavailable in clinical routine (13–23). Recently, the first *in vivo* oPs lifetime images were determined with the dedicated multi-photon J-PET scanner prototype (24), and notably also the first *in vivo* oPs lifetime measurements with a commercial PET/CT system were demonstrated (25, 26). Different dedicated image reconstruction techniques for oPs lifetime imaging have been presented in the literature (20, 22, 27–32).

The oPs lifetime can be measured by determining the time difference between a prompt-photon, emitted during the nuclear decay along with the positron, and the two photons with 511keV energy from the positron annihilation. The prompt-photon serves as the start time, while the detection of the annihilation photons sets the stop time. The two annihilation photons are also used to determine the place of annihilation (33). Histograming all measured time differences gives a Positron Annihilation Lifetime (PAL) spectrum that contains several components, including the oPs lifetime. The oPs lifetime is of particular interest, as it depends on the molecular structure of the surrounding matter (9, 10). oPs lifetime measurements require a positron-emitting radionuclide with prompt-photon emission, together with the possibility of detecting and localizing three-photon events^1^ (3γE). The detection of 3γE poses significant challenges, particularly in a clinical environment. Positron emission tomography (PET) systems are designed to detect photon pairs with 511keV energy. The detection of single-photon events with different energies is not part of the core design of clinical PET/CT scanners. Nonetheless, Ref. (34) presented the first use of a clinical PET/CT scanner for oPs lifetime measurements by extending the detection and processing capabilities to 3γE. An accurate measurement of oPs lifetime requires the detection of a substantial number of 3γE. The increased sensitivity of long-axial field-of-view (LAFOV) PET/CT systems (35–38) proved to be a key factor for oPs lifetime measurement on a commercial PET/CT system.

Radionuclides with prompt-photon emission are readily available in clinics, of which ^68^Ga labeled with [^68^Ga]Ga-PSMA-617 and [^68^Ga]Ga-DOTA-TOC is by far the most widely adapted. ^82^Rb and to some extent ^124^I are also used in clinical routine, which is why Refs. (24, 25) relied on ^68^Ga and ^82^Rb for *in vivo* measurements. The prompt-photon branching ratio (BR_γ_) is, of course, a key physical parameter to maximize the count statistics of 3γE. ^68^Ga and ^82^Rb have only a limited BR_γ_. If the positron emission fraction is taken into account, also the seemingly high BR_γ_ of ^124^I drops significantly. ^44^Sc, on the other hand, has a very high BR_γ_ in conjunction with a high positron fraction, which makes it a prime candidate for oPs lifetime imaging (38, 39). There is legitimate hope that ^44^Sc can overcome the challenge of detecting enough 3γE for a reliable determination of the useful lifetime of oPs (38).

Although ^44^Sc is not yet available in clinical routine, production routes, purification and labeling as well as first in-human studies have been reported in the literature (40–49). ^44^Sc can be paired with its therapeutic analog ^47^Sc for theranostic applications, enabling seamless transitions between diagnostic imaging and targeted therapy. Adding diagnostic information from oPs lifetime imaging could boost the tailored effectiveness of therapeutic applications with ^47^Sc, the $\beta^{-}$-emitting theranostic partner of ^44^Sc.

In this brief report, we investigate the properties of ^44^Sc for oPs lifetime imaging on a commercial LAFOV PET/CT. While Refs. (25, 34, 50) showed that ^124^I outperforms ^68^Ga and ^82^Rb in terms of 3γE count statistics, the current study investigates the performance of ^44^Sc with respect to oPs lifetime imaging and how it compares to ^124^I using the methodology described in Refs. (25, 34, 50).

## 2 Method

^44^Sc was produced at the Paul Scherrer Institute (PSI, Switzerland). The radionuclide production and post-irradiation processing at PSI have been established and are being further developed and optimized, as documented in Refs. (46, 51, 52). At Inselspital’s Department of Nuclear Medicine (Switzerland) a standard NEMA image quality phantom (Data Spectrum Corp.) without lung insert was filled with a total of 41.7MBq at scan time. The dose calibrator in the Department of Nuclear Medicine (VDC-405/VIK-202, Comecer, The Netherlands) was cross-calibrated with a ^44^Sc reference activity from PSI. Ref. (53) describes the calibration of PSI’s dose calibrator for ^44^Sc. The activity concentration in the six phantom spheres at scan time was 40.68kBq/mL while the background concentration was 3.90kBq/mL. The phantom was scanned for 20min in the so-called singles mode on a Biograph Vision Quadra (Siemens Healthineers, USA). Singles mode stores all single-crystal interactions into a list mode file. The sorting of 3γE is performed using the same prototype software as described in Refs. (25, 34, 50). The annihilation photon energy window is 476 to 546keV with a double coincidence time window of 4.2ns, while the prompt-photon energy window is 720 to 735keV, i.e., the last two energy bins. Apart from the time and energy window selection, a minimal distance of 30 crystals (equivalent to a 100 mm radius) is applied in order to control the ^176^Lu background (34). No reconstruction algorithm is applied, i.e., the spatial localization of the 3γE is purely based on time-of-flight (TOF) of the 511keV photons (34). As described in Ref. (34), Quadra resolves photon energies up to 726keV. Beyond this energy, all detected photons are collected in a single energy bin. Since the prompt-photon of ^44^Sc has an energy of 1157.022±0.015keV, all prompt-photon events are located in the last energy bin. The time differences between the annihilation and prompt-photons for each 3γE were binned in order to obtain a PAL spectrum. The time bins are 133ps wide. For the parameter fit we select only those 3γE with time differences between −2ns and 8.6ns.

For the determination of the oPs lifetime, we rely on the same Bayesian fitting procedure as in Refs. (25, 34, 50). The fit model for the PAL spectrum consists of three lifetime components, i.e., direct annihilation, para-positronium and oPs, convoluted with a Gaussian function that models the detection system. Solving the convolution integral analytically, the fit model can be written in terms of error functions:

$$F(\Delta t)=b+N\cdot \sum_{c=1}^{3}\frac{BR_{c}}{2\tau_{c}}e^{\left(\sigma^{2}-2\Delta t\;\tau_{c}+2\Delta_{0}\;\tau_{c})/(2\tau_{c}^{2}\right)}\;\cdot \mathrm{erfc}\left(\frac{\sigma }{\sqrt{2}\tau_{c}}+\frac{\Delta_{0}-\Delta t}{\sqrt{2}\sigma }\right).$$ (1)

In Equation 1, b denotes a constant background and N is a normalization constant. The relative branching ratios of the three lifetimes $\tau_{1,2,3}$ are $BR_{1,2,3}$. The two parameters σ and $\Delta_{0}$ define the Gaussian function. They represent the timing resolution and time offset. We use a Bayesian fitting procedure that minimizes a Gaussian likelihood for determining the parameter’s posterior distributions. Equation 2 shows the prior distributions for the fit parameters

$$\begin{array}{cc} & \tau_{3}∼N(1.78\;\text{ns},0.8\;\text{ns}),\\ & BR_{1,2,3}∼\mathrm{Dirichlet}(0.75,3.1,1.15),\\ & \sigma ∼N(0.1\;\text{ns},0.05\;\text{ns})\;,\\ & \Delta ∼N(0\;\text{ns},0.5\;\text{ns}),\\ & N∼N(A,0.1\cdot A), \end{array}$$ (2)

where A is the integrated of the PAL spectrum with a subtracted background b. The value of b is determined as the mean counts with time differences smaller than −2.7ns. The values of the direct annihilation and oPs lifetime are fixed to reference values of $\tau_{1}=0.388\;\text{ns}$ and $\tau_{2}=0.125\;\text{ns}$. Setting priors for $\tau_{1,2}$ does not impact the result significantly (25, 34). The Bayesian approach allows us to marginalize nuisance parameters. In fact, we are mostly interested in $\tau_{3}$ and the branching ratios (for sanity checks and comparison with established results from the literature). We report the fit results in terms of marginalized posterior distributions. The posterior distribution for $\tau_{3}$ is almost a perfect Gaussian function, hence the standard deviation is a reasonable measure for the uncertainty. However, this does not apply to $BR_{1,2,3}$ and we therefore provide the highest density interval (HDI) of the posterior distribution in the results.

We determined the oPs lifetime for the six spheres $s_{1\cdots 6}$ of the NEMA phantom (nominal diameters: 10, 13, 17, 22, 28, 37mm). Furthermore, we binned the spatial distribution of the detected 3γE into voxels of $4\times 4\times 4\;{\text{mm}}^{3}$. For each voxel, the oPs lifetime is determined according to the same Bayesian fitting as for the phantom spheres.

## 3 Results

**Figure 1:**
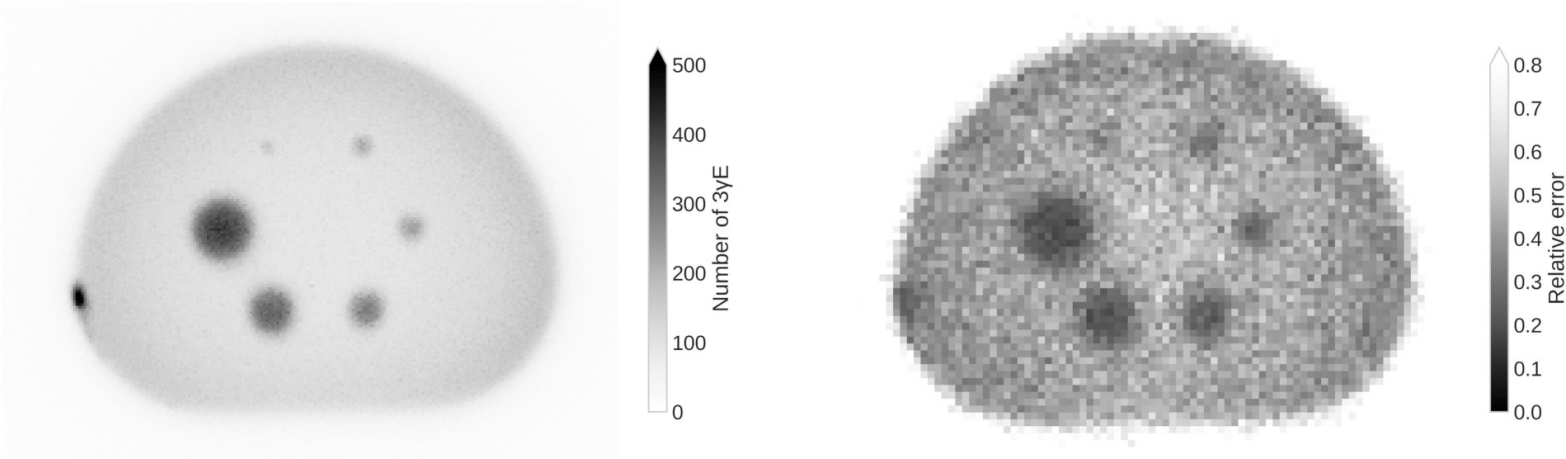
MIP of the 3γE histoimage with a voxel size that corresponds to the CT image (left) and the relative error in the background region of the PAL spectrum in a single slice with $4\times 4\times 4\;{\text{mm}}^{3}$ voxel size (right).

The left panel of Figure 1 shows the maximum intensity projection (MIP) of the 3γE histoimage. The binning is chosen according to the CT image, i.e., $1.52\times 1.52\times 1.65\;{\text{mm}}^{3}$. Even without any reconstruction methodology, i.e., using only TOF for the localization of the 3γE, the smallest sphere $s_{1}$ of the NEMA phantom is visible. The absence attenuation correction is clearly visible through the darkening on the border of the phantom. Some ^44^Sc activity stuck to the left wall of the phantom.

The total number of 3γE in the full field of view collected during the 30 min scan is 539862149 for a triple coincidence time window from −15ns to +15ns. These are, however, mostly random 3γE. In contrast, a 20 min scan in standard coincidence mode with a larger coincidence window of 435keV to 585keV of the same phantom yields 2 405 451 960 net trues. This includes the standard random correction methods for coincidence PET.

On the right of Figure 1 the relative error in the background region of the PAL spectrum, i.e., for time differences that are smaller than −2.7ns, is shown. The error inside the spheres decreases as there is a higher activity concentration. Due to the decreasing number of 3γE towards the center of the phantom, the error increases towards the center of the phantom (there is no attenuation correction).

**Figure 2:**
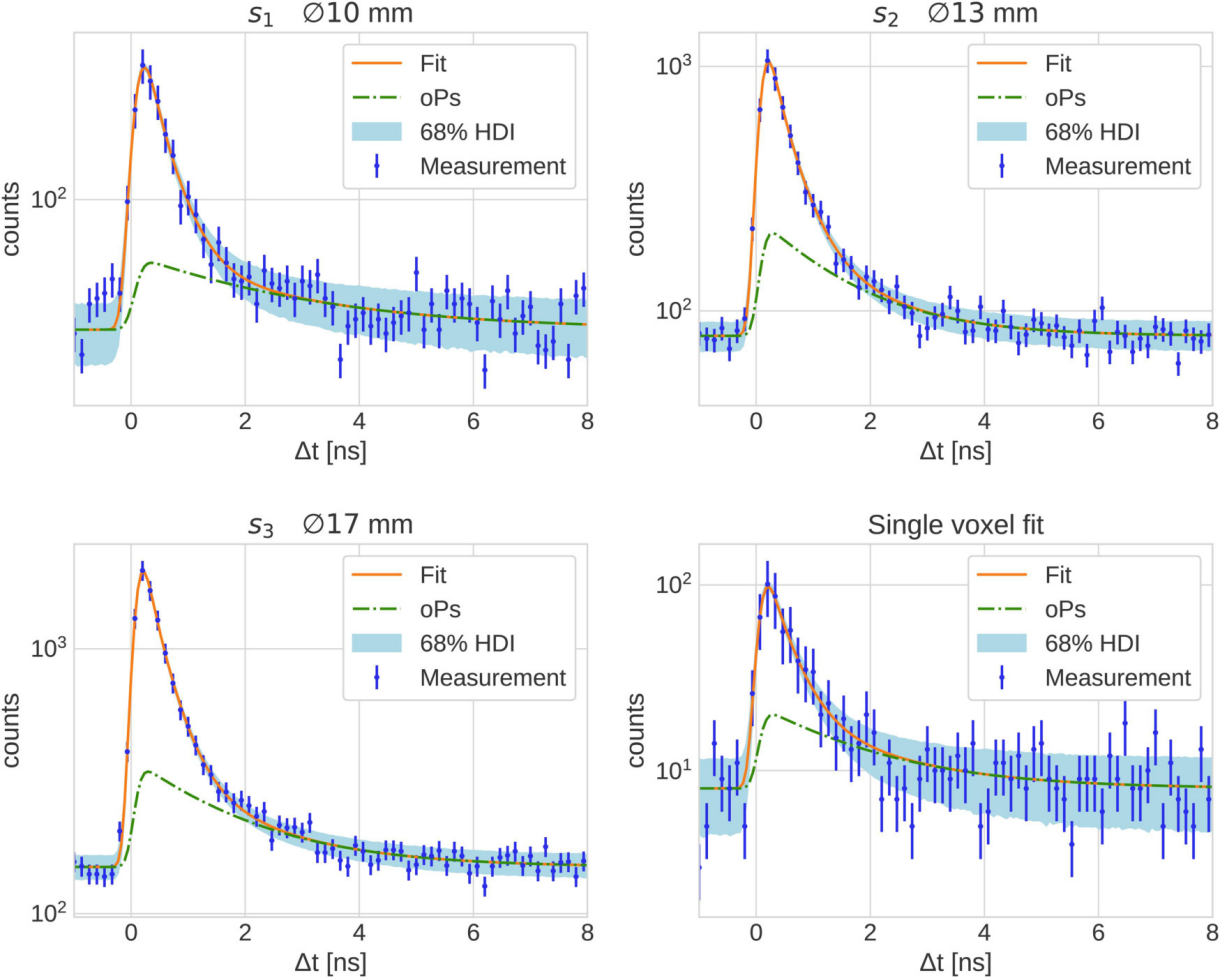
PAL spectrum of all 3γE with the fit prediction in the three smallest spheres of the NEMA phantom and of a single $4\times 4\times 4\;{\text{mm}}^{3}$ voxel in the center of $s_{6}$.

Figure 2 shows the measured PAL spectrum with the fit prediction for the three smallest spheres and a single voxel in the center of the largest sphere $s_{6}$. The error bars plotted on the measurement points are the relative error in the background region of the PAL spectrum, i.e., the relative standard deviation of all time differences <−2.7ns. The 68% HDI plotted in Figure 2 represents prediction uncertainty of the fit. The fit results corresponding to the PAL spectrum in Figure 2 are reported in Table 1 together with the fit results of the larger phantom spheres. The posterior distribution of $\tau_{3}$ is Gaussian, hence we report the error on $\tau_{3}$ as a standard deviation in Table 1. This does not apply to the relative branching ratios of the three lifetime components $BR_{1,2,3}$, since these are Dirichlet distributed random variables. Their error is therefore quoted as a 68% HDI.

**Table 1:** Fit results for the six phantom spheres and a single $4\times 4\times 4\;{\text{mm}}^{3}$ voxel in the center of $s_{6}$.

Fit	$\tau_{3}\;[\text{ns}]$	$BR_{1}$	${\text{HDI}}_{BR_{1}}$	$BR_{2}$	${\text{HDI}}_{BR_{2}}$	$BR_{3}$	${\text{HDI}}_{BR_{3}}$
$s_{1}\emptyset 10\;\text{mm}$	2.65±0.50	0.072	[0.0, 0.091]	0.659	[0.608, 0.736]	0.269	[0.242, 0.301]
$s_{2}\emptyset 13\;\text{mm}$	1.39±0.20	0.077	[0.049, 0.106]	0.623	[0.573, 0.679]	0.30	[0.267, 0.324]
$s_{3}\emptyset 17\;\text{mm}$	1.76±0.18	0.062	[0.041, 0.083]	0.651	[0.62, 0.687]	0.287	[0.27, 0.301]
$s_{4}\emptyset 22\;\text{mm}$	1.86±0.09	0.057	[0.047, 0.067]	0.655	[0.639, 0.671]	0.288	[0.281, 0.296]
$s_{5}\emptyset 28\;\text{mm}$	1.73±0.1	0.091	[0.08, 0.103]	0.603	[0.585, 0.622]	0.306	[0.296, 0.314]
$s_{6}\emptyset 37\;\text{mm}$	1.78±0.08	0.066	[0.057, 0.076]	0.642	[0.627, 0.657]	0.292	[0.285, 0.299]
Voxel	1.79±0.57	0.051	[0.0, 0.063]	0.609	[0.553, 0.717]	0.34	[0.266, 0.386]

**Figure 3:**
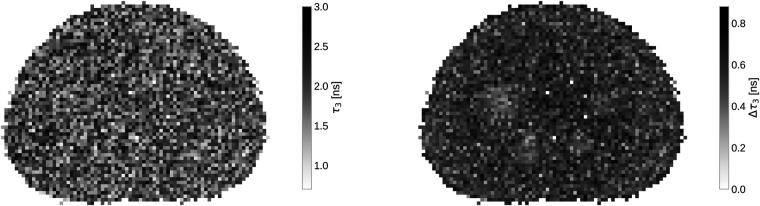
Slice of the oPs lifetime image (left) and $\tau_{3}$ error (right) with $4\times 4\times 4\;{\text{mm}}^{3}$ voxels.

In Figure 3 a slice of the full oPs lifetime image, together with the fit error on $\tau_{3}$ with a $4\times 4\times 4\;{\text{mm}}^{3}$ binning, is presented. While the oPs lifetime image is not particularly interesting - after all, the phantom is filled with water - the marginalized uncertainty on $\tau_{3}$ clearly increases in the central region of the phantom. Note that only for the four largest spheres, the error decreases visibly.

## 4 Discussion

**Figure 4:**
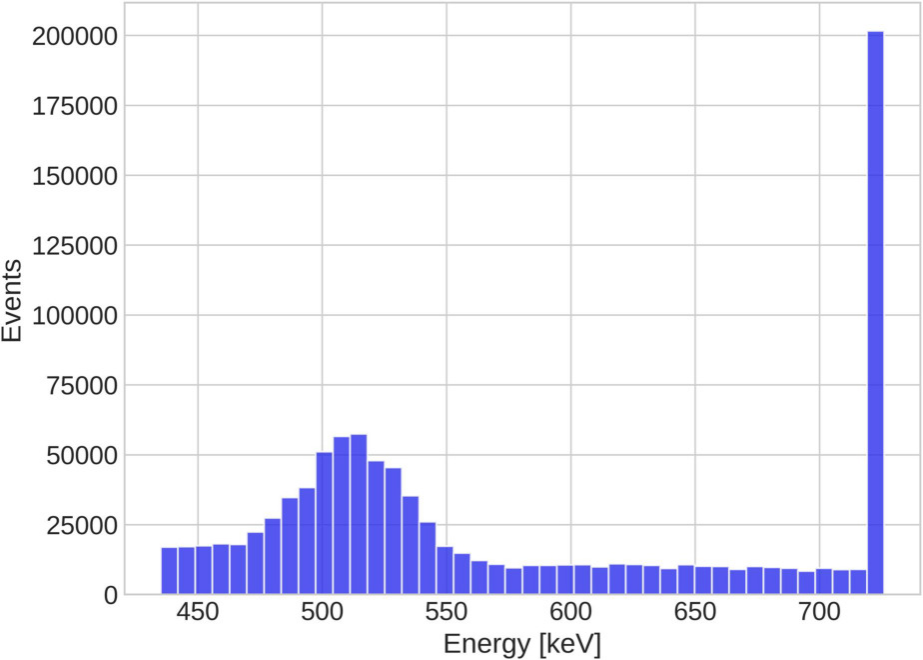
Energy spectrum of $10^{6}$ detector hits from ^44^Sc.

From the discussion in Ref. (34), it is clear that the key question is whether the high BR_γ_ of ^44^Sc can overcome the Quadra’s inability to resolve ^44^Sc’s photopeak. Detector hits above 726keV are collected in a single integrating bin, as clearly illustrated in Figure 4. One should, therefore, expect that more random coincidences are selected due to the high prompt-photon energy of ^44^Sc. The right panel of Figure 3 already hints towards a high random 3γE rate: even inside the spheres, the relative error in the background region of the PAL spectrum exceeds 20%. For a comparison, Ref. (50) only considered those voxels with less than 20% background error for oPs lifetime imaging.

The large number of random 3γE is reflected in the statistical uncertainty of $\tau_{3}$ reported in Table 1. All values for $\tau_{3}$ in the phantom are consistent with the literature value of 1.839±0.015ns for water from Ref. (54) and with the results from Ref. (50) within their statistical uncertainty [note also the reference values in Ref. (17)]. However, the marginalized uncertainties reported in Table 1 are rather large: only starting from $s_{3}$ the relative error starts dropping below 10% (and reaches even 31.9 % in a single voxel). This is likely more than the precision required to sense different oxygenation levels in lesions, as discussed in Ref. (16).

$\tau_{3}$’s uncertainty is seen in Figure 3 as well. The variation on $\tau_{3}$ across the whole phantom is quite large, given that the expected oPs lifetime should be the same across the whole phantom. In the right panel of Figure 3, only very few voxels have an error below 0.3ns. The mean uncertainty on $\tau_{3}$ across the slice shown in Figure 3 is 0.53ns. Only the four largest spheres of the phantom have a visibly smaller uncertainty compared to the phantom background.

The fit of the oPs lifetime critically depends on the time differences after the peak in the PAL spectrum, i.e., on values close to the random 3γE background. A useful quantity to characterize the 3γE count statistics is therefore the peak signal-to-background ratio (pSBR) in a PAL spectrum. In the measurements with ^124^I, Ref. (50) reported a pSBR of about 55.5 for a $4\times 4\times 4\;{\text{mm}}^{3}$ voxel in the water tube with an activity concentration of 252kBq/ml and a scan time of 15min. For the PAL spectrum in the $4\times 4\times 4\;{\text{mm}}^{3}$ voxel in Figure 2, however, the pSBR is only about 12.6. Despite the activity concentration being higher in the ^124^I measurements of Ref. (50), the scan duration is 5min shorter. The error on $\tau_{3}$ in a single voxel (last row in Table 1) is about four times larger than the error reported in Ref. (50) for the same voxel size. A similar picture arises when looking at volumes of similar size, e.g., the sphere $s_{4}$ has a volume of 5.57mL and is comparable with the volume of the tubes in Ref. (50). The relative error on $\tau_{3}$, however, is 4.8% while Ref. (50) reports a 1.1% error for a 5mL tube with water. This comparison is even more striking, when considering the BR_γ_ per positron, which is almost 8 times higher for ^44^Sc than for ^124^I. With the given methodology, resolving the photopeak therefore seems key for a low random 3γE rate. ^44^Sc’s high BR_γ_ cannot overcome Quadra’s limited detection capabilities for high-energy photons. Given the energy spectrum in Figure 4, it is clear that extending the prompt-photon energy window does not yield a significant reduction of random 3γE. Also, note that ^124^I’s lower prompt-photon energy (almost half compared to ^44^Sc) increases the probability to interact within the detector crystals. It should be emphasized that this conclusion applies to the given methodology. Different detection methods (24) or event selection procedures and/or random 3γE estimations as e.g., in Ref. (55) may reduce the uncertainties on $\tau_{3}$ in the case of high-energy prompt-photons. We leave such an investigation for future studies.

Ref. (56) did not attempt to perform a voxel-wise fit nor a fit to the three smallest spheres of the NEMA phantom. On the other hand, Ref. (57) seems to be able to fully exploit the high prompt-photon BR_γ_ of ^44^Sc. Both scanners in these studies do not suffer from the limited energy range of Quadra and the event selection and reconstruction algorithms are different.

In contrast to ^44^Sc, ^43^Sc’s prompt photon is within Quadra’s energy range and therefore, the afore mentioned discussion of the high-energy prompt-photons does not apply. However, the BR_γ_ per positron is in the same order of magnitude as ^124^I and ^82^Rb i.e., much lower than for ^44^Sc.

## 5 Conclusions

Given Quadra’s limited energy resolution and the current methodology for selecting 3γE, it does not seem that ^44^Sc is able to outperform ^124^I in terms of count statistics for oPs lifetime imaging, despite its favorable physical properties and clinical prospects.

## Data Availability

The raw data format is not publicly available. Evaluated data are available upon reasonable request. Requests to access the datasets should be directed to lorenzo.mercolli@insel.ch.

## Footnotes

1 In this study, we do not consider three-photon decays of oPs.
